# Molecular and Cellular Mechanisms for Proteinuria in Minimal Change Disease

**DOI:** 10.3389/fmed.2018.00170

**Published:** 2018-06-11

**Authors:** Roberta Bertelli, Alice Bonanni, Gianluca Caridi, Alberto Canepa, G. M. Ghiggeri

**Affiliations:** ^1^Laboratory of Molecular Nephrology, Genoa, Italy; ^2^Nephrology, Dialysis, Transplantation Unit, Integrated Department of Pediatrics and Hemato-Oncology Sciences, Istituto Giannina Gaslini IRCCS, Genoa, Italy

**Keywords:** minimal change disease, nephrotic syndrome, proteinuria, experimental models, focal segmental glomerulosclerosis

## Abstract

Minimal Change Disease (MCD) is a clinical condition characterized by acute nephrotic syndrome, no evident renal lesions at histology and good response to steroids. However, frequent recurrence of the disease requires additional therapies associated with steroids. Such multi-drug dependence and frequent relapses may cause disease evolution to focal and segmental glomerulosclerosis (FSGS) over time. The differences between the two conditions are not well defined, since molecular mechanisms may be shared by the two diseases. In some cases, genetic analysis can make it possible to distinguish MCD from FSGS; however, there are cases of overlap. Several hypotheses on mechanisms underlying MCD and potential molecular triggers have been proposed. Most studies were conducted on animal models of proteinuria that partially mimic MCD and may be useful to study glomerulosclerosis evolution; however, they do not demonstrate a clear-cut separation between MCD and FSGS. Puromycin Aminonucleoside and Adriamycin nephrosis are models of glomerular oxidative damage, characterized by loss of glomerular basement membrane polyanions resembling MCD at the onset and, at more advanced stages, by glomerulosclerosis resembling FSGS. Also Buffalo/Mna rats present initial lesions of MCD, subsequently evolving to FSGS; this mechanism of renal damage is clearer since this rat strain inherits the unique characteristic of overexpressing Th2 cytokines. In Lipopolysaccharide nephropathy, an immunological condition of renal toxicity linked to B7-1(CD80), mice develop transient proteinuria that lasts a few days. Overall, animal models are useful and necessary considering that they reproduce the evolution from MCD to FSGS that is, in part, due to persistence of proteinuria. The role of T/Treg/Bcells on human MCD has been discussed. Many cytokines, immunomodulatory mechanisms, and several molecules have been defined as a specific cause of proteinuria. However, the hypothesis of a single cell subset or molecule as cause of MCD is not supported by research and an interactive process seems more logical. The implication or interactive role of oxidants, Th2 cytokines, Th17, Tregs, B7-1(CD80), CD40/CD40L, c-Mip, TNF, uPA/suPAR, Angiopoietin-like 4 still awaits a definitive confirmation. Whole genome sequencing studies could help to define specific genetic features that justify a definition of MCD as a “clinical-pathology-genetic entity.”

## MCD, more than a “clinical –pathology entity”

### Definition of MCD

Minimal Change Disease (MCD) is a clinical condition characterized by heavy proteinuria and nephrotic syndrome that classically arises acutely in patients aged under 18 years. Histologically, immunofluorescence staining is typically negative, even though MCD is characterized by fusion of foot process in podocytes that can be detected only by Electron Microscopy. Concerning therapy, most MCD patients respond to steroids that usually reverse the clinical picture in a few days. Steroid dependence is also frequent and, when present, it requires additional therapy. Besides steroids, the natural history of MCD is influenced by other drugs that are essential to obtain a good stability over months/years allowing withdrawal of the initial therapy. Renal histology justifies the definition of “minimal changes” and the good sensitivity to drugs supports the concept that MCD is a benign condition.

### Overlaps between MCD and FSGS

As regards MCD, there is not a complete correspondence between sensitivity to steroids and pathology findings, since not all patients respond to steroids, while a minority of patients responding to steroids present sclerotic lesions that involve focal segments of glomeruli and are associated with diffuse fusion of foot process. When these pathological features are well structured, then the condition is defined as primary Focal Segmental Glomerulosclerosis (FSGS), that represents a separate entity with different clinical and prognostic features (Table 1). Moreover, there are conditions in which the clinical-pathology trait is less definite and renal lesions evolve from initial pathological findings of minimal lesions to FSGS. Reduced responsiveness to therapies often characterizes these patients who usually require the association of steroids and calcineurin inhibitors. The existence of clinical and pathology overlaps between MCD and FSGS supports the definition of MCD as not a completely separate entity, which may evolve into FSGS over years. This suggests that MCD and FSGS could represent, in a proportion of patients, two phases of the same disease (1, 2). However, the actual link between MCD and FSGS is still unclear (3, 4). A correct definition of MCD should be based on the observation of the concomitant absence of histological lesions and a good clinical response to steroids in the same patient.

**Table 1:** Clinical features of Minimal Change disease (MCD) and Focal Segmental Glomerulosclerosis (FSGS).

	**MCD**	**FSGS**
Sex prevalence	No	No
Age prevalence	Yes	No
Secondary forms	Rare	Frequent
Mendelian traits	Rare	Frequent
Histology	No lesions	Focal-segmental
IF	Negative	Positive
EM	Foot processes effacement	Foot processes effacement
Therapy sensitivity		
-Steroids	Frequent	Rare
-Calcineurin inhibitors	Frequent	Possible
-Rituximab	Frequent	Rare
Progression to CRF	Rare	Frequent

## Genetics of MCD and overlaps with FSGS

While a genetic origin is frequent in FSGS and, more in general, in nephrotic syndrome not responding to any therapy, genetic data in MCD are limited and their interpretation should consider the discrimination between MCD and FSGS. Genetic studies could offer more solid criteria for discriminating between the two conditions.

Familial aggregation and cases with siblings or parent-child aggregation were described for MCD (5), which suggests a possible genetic origin of the disease. Structured studies on genetics of MCD are now in progress and some interesting associations have been reported. In the case of FSGS, mutations in several genes causative of the disease were detected in the last decade (Table 2). MCD is considered a disease with complex inheritance where HLA genes are susceptibility loci (6–9) and inflammatory triggers, or modifiers of the genetic milieu, act as second hit. In general, there are ethnic trends according to which MCD (or better saying steroid sensitive nephrotic syndrome) is more frequent in Asia and Asian children have a seven-fold risk of developing the disease compared to non-Asian races (10). Variants of the HLA-DQA1 and of other MHC loci, associated with other gene variants (*PLCG2*) (11) and/or with environmental factors should contribute as second hits to the pathogenesis (12). It is of interest that a model implying the co-existence of HLA and other genes or molecules has been proposed also for other glomerular diseases such as IgA and membranous nephropathy (13). We expect some evolution from whole genome sequencing and it is probable that, in a near future, MCD will be defined also on the basis of specific genetic features.

**Table 2:** Genes associated to MCD and FSGS.

**Gene**	**Description**	**Acc. Number**	**Transmission**
ACTN4	Actinin, alpha 4	NM_004924.4	AD
ADCK4	AarF domain containing kinase 4	NM_024876.3	AR
ANLN	Anillin, actin binding protein	NM_018685.2	AD
ARHGAP24	Rho GTPase-activating protein 24	NM_001025616.2	AD
ARHGDIA	Rho GDP dissociation inhibitor (GDI) alpha	NM_001185078.1	AR
AVIL	Advillin	NM_006576.3	AR
CD2AP	CD2-associated protein	NM_012120.2	AR
CFH	Complement factor H	NM_000186.3	AR
COL4A3	Collagen type 4 alpha 3 chain	NM_000091.4	AR/AD
COL4A4	Collagen type 4 alpha 4 chain	NM_000092.4	AR/AD
COL4A5	Collagen type 4 alpha 5 chain	NM_000495.4	X-Linked
COQ2	Coenzyme Q2 4-hydroxybenzoate polyprenyltransferase	NM_015697.7	AR
COQ6	Coenzyme Q6 mono-oxygenase	NM_182476.2	AR
CRB2	Crumbs homolog 2	NM_173689.5	AR
CUBN	Cubilin (intrinsic factor-cobalamin receptor)	NM_001081.3	AR
DGKE	Diacylglycerol kinase, epsilon	NM_003647.2	AR
EMP2	Epithelial membrane protein 2	NM_001424.4	AR
FAT1	FAT tumor suppressor homolog 1	NM_005245.3	AR
INF2	Inverted formin, FH2 and WH2 domain containing	NM_022489.3	AD
ITGA3	Integrin, alpha 3 (antigen CD49C, alpha 3 subunit of VLA-3 receptor)	NM_005501.2	AR
ITGB4	Integrin, beta 4	NM_000213.3	AR
KANK1	KN motif and ankyrin repeat domain containing protein 1	NM_001256876.1	AR
KANK2	KN motif and ankyrin repeat domain containing protein 2	NM_015493.6	AR
KANK4	KN motif and ankyrin repeat domain containing protein 4	NM_181712.4	AR
LAGE3	L antigen family member 3	NM_006014.4	AR
LAMA5	Laminin alpha-5	NM_005560.4	AR
LAMB2	Laminin, β2	NM_002292.3	AR
LMX1B	LIM homeobox transcription factor 1, beta	NM_00117414.1	AD
MAGI2	membrane associated guanylate kinase, WW and PDZ domain containing 2	NM_001301128.1	AR
MTTL1	Mitochondrially encoded tRNA leucine 1	NC_012920.1	AR
MYH9	Myosin heavy chain 9	NM_002473.4	AD
MYO1E	Homo sapiens myosin IE (MYO1E)	NM_004998.3	AR
NPHS1	Nephrin	NM_004646.3	AR
NPHS2	Podocin	NM_014625.2	AR
NUP107	Nucleoporin 107 kDa	NM_020401.2	AR
NUP205	Nucleoporin 205 kDa	NM_015135.2	AR
NUP93	Nucleoporin 93 kDa	NM_014669.3	AR
OSGEP	O-sialoglycoprotein endopeptidase	NM_017807.3	AR
PDSS2	Prenyl (decaprenyl) diphosphate synthase, subunit 2	NM_020381.3	AR
PLCE1	Phospholipase C, epsilon 1	NM_016341.3	AR
PTPRO	Protein tyrosine phosphatase, receptor type, O	NM_030667.2	AR
SCARB2	Scavenger receptor class B, member 2	NM_005506.3	AR
SGPL1	sphingosine-1-phosphate lyase 1	NM_003901.3	AR
SMARCAL1	SWI/SNF-related, matrix-associated, actin-dependent regulator of chromatin, subfamily a- like 1	NM_014140.3	AR
TP53RK	TP53 regulating kinase	NM_033550.3	AR
TPRKB	TP53RK binding protein	NM_001330386.1	AR
TRPC6	Transient receptor potential cation channel, subfamily C, member 6	NM_004621.5	AD
WDR73	WD repeat domain 73	NM_032856.2	AR
WT1	Wilms tumor 1	NM_024426.4	AD
XPO5	Exportin 5	NM_020750.2	AR

MCD as a part of a syndrome has been uniquely reported in association with *EXT1* mutations that cause autosomal dominant exostoses (OMIM 133700) (14). Isolated MCD with autosomal recessive inheritance has been reported in association with mutations of *EMP2* gene encoding a regulatory protein of caveolin-1, that is expressed in podocytes and in renal endothelium (15). Rare cases of MCD associated with mutations of *KANK1* and *KANK2* genes have also been described in two families with a mixed pattern of MCD and FSGS (16). Given the similarities between MCD and FSGS, we cannot exclude that mutations in one of the recognized gene panels reported for FSGS may coexist in MCD. An example is NPHS1 mutation, that is causative of one of the most clinically relevant forms of congenital nephrotic syndrome. Three cases of NPHS1 mutation in children with nephrotic syndrome responsive to steroids and cyclosporine have been described, suggesting that hypomorphic mutations (such as those here reported) may coexist with the more frequent and severe clinical phenotypes (17).

## Mechanisms 1. clinical evidence in MCD

### What we learned from clinical associations

There are cases of secondary MCD for which a direct relationship with a definite trigger or cause has been hypothesized. Cases of an association with drugs such as salazopyrin, D-penicillinamine, mercury, and gold have been reported, in which proteinuria remits following their withdrawal. In this case, the conclusion is that simple molecules may modify podocyte structure inducing a reversible defect that underlies the transitory nature of foot process fusion phenomenon. MCD may occur in parallel with major blood disorders such as Hodgkin (18, 19) and non-Hodgkin lymphoma (20–22) (mainly marginal zone B-cell lymphoma and chronic lymphocytic leukemia) and other lymphoproliferative disorders (such as multiple myeloma) and cancer (18, 23) (i.e., thymoma, bronchogenic cancer, and colon cancer). MCD is the most frequent glomerular disease associated with Hodgkin lymphoma (19, 24). C-Mip overexpression in tumor cells and in podocytes is a molecular signature of the association (25), that suggests a pathogenetic implication (for c-Mip see below). The association with non-Hodgkin lymphoma has been less frequently described; Kofman et al. (22) studied a large series of 13,992 patients and reported on 18 cases with this association, 50% of them presenting marginal B cell lymphoma or chronic lymphocytic leukemia. These are very rare associations in which the nephrotic syndrome generally remits after resolution of the primary problem.

The association of MCD with multiple myeloma is also rare. In myeloma, immunoglobulins or immunoglobulin fragments such as light chains present structural modifications that alter their molecular charge, often in terms of cationization and glycosylation. Cationic and/or hyper-glycosylated immunoglobulins are potential pathological effectors of proteinuria. Studies performed several decades ago clearly demonstrated that the charge of molecules is potentially able to modify the mechanism of repulsion of proteins at the glomerular level that, for molecules with a molecular mass >46 KDa, is mainly based on their charge (26, 27). Experimental models of proteinuria induced by infusion of cationic proteins strongly support this concept, since these molecules are not repulsed by glomerular polyanions and are freely excreted into urines. The possibility that a modification of protein charges could induce MCD has been explored in the past, without consistent results. There is now an increasing interest in the role of IgM glycosylation, that is now under study and could lead to more structured conclusions.

### What we learned from specific treatments of MCD

One of the clinically relevant characteristics of MCD is that it has been successfully treated from many years in spite of lack of any knowledge about mechanisms. Cyclosporine had historically a main role in successful treatment of MCD dependent and/or resistant to steroids (28). Cyclosporine is an inhibitor of calcineurin that inhibits, in turn, a nuclear factor in T lymphocytes. It was only after the study by Faul et al. (29) that it became clear that cyclosporine may also have a direct effect on podocytes, which blunts its importance as immunosuppressor. It blocks, in fact, the calcineurin dependent dephosphorylation of synaptopodin in podocytes and this protects the enzymatic degradation of synaptopodin by cathepsin L: the final result is the stabilization of the cytoskeleton. The study above also showed that the cytoskeleton in podocytes is crucial for their stability and that its perturbation leads to proteinuria. Molecular studies on genetic inherited nephrotic syndrome showing mutations in genes encoding cytoskeleton proteins confirmed this key function (30).

## Mechanisms 2. cell studies

### T-effectors

The possible implication of T cell effectors in MCD is mainly supported by the finding that supernatants of T cell hybridoma deriving from blood of patients with MCD determine foot process effacement and proteinuria in rats (31). This is a strong observation dating back to 1991; since then, no new evidence on specific proteinuric factors deriving from the T hybridoma was added to the original report, leaving this study field open to speculation and evolution. The existence of an imbalance in T cell subsets, with predominance of CD8+ over CD4+ and an increase in Natural Killer (NK) cells was the main hypothesis in MCD pathogenesis until the early 2000s (32). However, it is also clear that a single cell compartment as cause of MCD is not supported by research. The overview on circulating cells and cytokines potentially implicated in MCD that is reported below will give an idea of the complexity of the pathogenesis. Two specialized T cell lineages potentially involved in MCD are Th2 and Th17 (33–35). Th2 role is supported by the observation of a specific cytokine profile in patients with MCD (i.e., upregulation of IL4, IL5, IL9, IL10, IL13) (34, 36–38). Furthermore, Buffalo/Mna rats were characterized by Th2 polarization and presented a predominant increase in IL4 and IL13 levels, preceding the development of nephrotic syndrome (39, 40). A selective IL13-Th2 profile characterized children with nephrotic syndrome, who also presented upregulation of IL13 (but not IL4) mRNA by CD4+ and CD8+ cells during relapse (34). On the other hand, it is known that IL13 overexpression may directly induce proteinuria and podocyte lesions in rats (41) (see below). Th17 cells derive from naïve CD4+ upon stimulation with IL6 and IL13 (42). Th17 levels rise in several glomerulonephritides characterized by inflammatory glomerular lesions. This is not observed in MCD and raises doubts on a selective role of Th17 in renal disease (42). In general, previous studies focusing on specific cytokines reached fragmentary conclusions and the interpretation of their results must take into account that different technologies were used in the separation of cellular subsets and in cytokine determination. For this reason, IL8 and IL12 were reported both as high and low by different Authors and other cytokines such as IL10, IL2, IL4, and IL6 were described as low in serum despite their synthesis by purified polymorphonucleates and monocytes was high.

### T regulatory cells

T regulatory cells (Tregs) are a dynamic cell population: at equilibrium, their level is low but they are rapidly generated from CD4 T cells, that expand in response to T cell Receptor (TCR) stimulation by class II HLA peptides. CD4 T cells differentiate into Tregs (CD4+ CD25+ Foxp3+) or into T effector cells (CD4+ CD25+ Foxp3−), depending on the cytokine milieu [for a review see Josefowicz et al. (43)]. Since Tregs and Teffs have anti-inflammatory and pro-inflammatory effects, respectively, it is clear that the balance between Tregs and Teffs derived from CD4+ has a crucial role in adaptive response.

**Figure 1:**
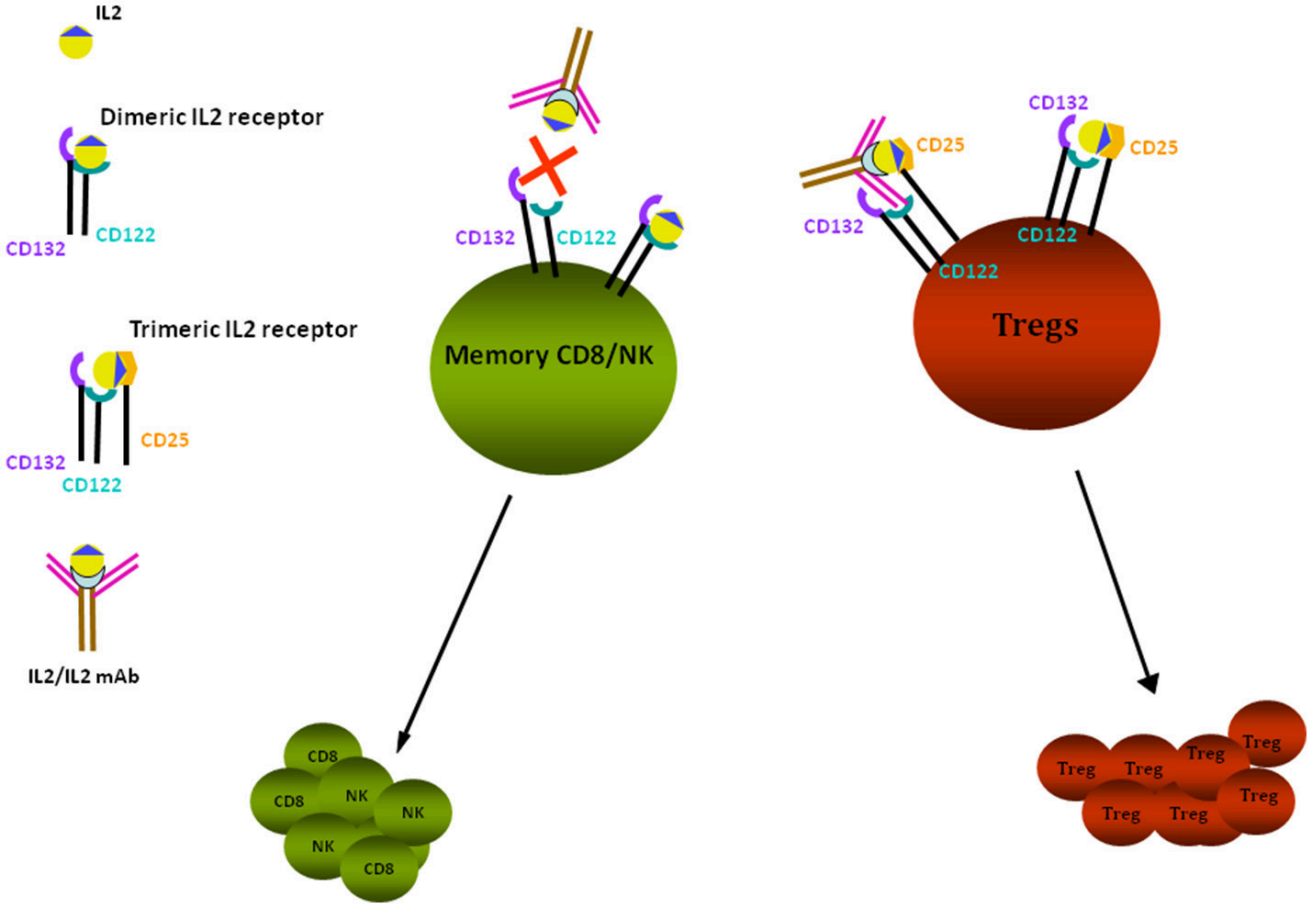
IL2 effects on Tregs. Tregs proliferation is stimulated through binding of IL2/anti-IL2 Ab to the trimeric IL2 receptor composed by CD122-CD132-CD25 subunits, having high affinity for the IL2 and IL2-anti-IL2 complex; the high affinity receptor is expressed by CD4^+^ cells, including Tregs. Low affinity receptors are expressed by memory CD8^+^ and NK cells: they are composed of the two subunits CD122 and CD132 that bind free IL2. In presence of anti-IL2Ab, the free quota of IL2 is decreased and proliferation of CD8+/NK is reduced. Tregs are dependent from IL2 for their survival and proliferation. *In vivo* administration of IL2 and of IL2 coupled to a monoclonal anti-IL2 antibody (JES6-1), specific for the CD132 subunit, prevents the activation of CD8^+^ and NK cells and allows the selective stimulation and proliferation of Tregs by the interaction with CD25 receptor subunit.

Several studies in experimental models support the association between low Tregs and proteinuria [for a review see Bertelli et al. (44)]. Depending on the timing of the observation, animals expressing low Treg titers may develop alternatively MCD or FSGS. The hypothesis of an association between low Tregs and proteinuria has been confirmed in almost all models of proteinuria. In particular, (i) Adriamycin nephrosis can be modulated by infusing Foxp3-transduced T cells and/or by infusion of adenosine and/or by administration of low dose IL2 (45, 46); in all these cases, circulating Tregs were upregulated and proteinuria downmodulated; (ii) direct infusion of Tregs into Buffalo/Mna rats (40) was associated with proteinuria reduction and regression of renal lesions; (iii) Tregs role has also been investigated in LPS nephropathy that represents, as already described, a model of transient proteinuria; in this case, Tregs level was modulated by the administration of IL2/anti-IL2 immunocomplexes (Figure 1), that induces differentiation of CD4+ cells into Tregs (47). Polhill et al. (46) showed that infusion of IL2/anti-IL2 can reduce proteinuria and improves renal function in rats with Adriamycin nephrosis. In mice treated with LPS, Bertelli et al. (47) obtained a reduction of proteinuria utilizing the same therapeutic approach, but results were less clear and were inconclusive regarding the renal outcome since IL2 did not modify renal lesions.

Evidence for a role of Tregs in human MCD is scanty but deserves a careful analysis in view of the possibility that these cells may be modulated *in vivo*. An interesting starting point is the association of MCD with IPEX (48), a syndrome characterized by immunodeficiency, polyendocrinopathy, and enteropathy, caused by a genetic mutation of FOXP3 that inactivates Tregs. It is of interest that, in a child with IPEX, nephrotic syndrome disappeared after bone marrow transplantation that normalized Treg functions (48). Studies in human pathology demonstrated that circulating levels of Tregs were low in patients with active MCD (49) and that low Treg levels correlated with high oxidant production deriving from ATP in polymorphonucleates. The balance between ATP and ADP is crucial to oxidant generation by circulating cells and apyrase has a key role in it. Apyrase is, in fact, the enzyme that reduces ATP and, as a consequence, it reduces oxidants. Studies by Bertelli et al. (49) produced evidence about the link among Tregs, apyrase, ATP, and oxidant generation in MCD. Modulation of Treg levels has become an interesting new potential treatment of MCD following the observation that low dose recombinant IL2 is safe and can be utilized in humans. In two important studies, low dose recombinant IL2 was utilized in patients with HCV-induced vasculitis and in Graft versus Host disease, showing that modulation of Treg levels was obtained in both cases (50, 51). So far, the unique pilot study in patients with renal diseases utilizing low dose recombinant IL2 was conducted in 4 patients with FSGS and nephrotic syndrome resistant to drugs (52). These patients were treated with six cycles of five IL2 infusions (1 × 10^6^ U/m2 1st month, 1.5 × 10^6^ U/m2 subsequent months) and had a stable 4- to 10-fold increment of Treg levels. Proteinuria, however, remained unchanged over 6 months, with no effects of IL2 at least in this set of patients. Good safety and tolerability of the drug suggested that low dose IL2 could be extended to MCD. However, given the better efficacy and tolerability of other drugs (i.e., anti-CD20) compared to IL2 (see below), there seems to be no reason to design a new study in this class of patients.

### B cells

This is a field of recent interest. In the last few years, anti-CD20 monoclonal antibodies (Rituximab) were successfully utilized in MCD (53), representing at this time the most effective treatment of MCD patients with steroid dependence (54). Anti-CD20 are not synonymous of B cell inhibitors, since it has been shown that, within *in vitro* podocytes, these antibodies bind to Sphingomyelin Phosphodiesterase acid-like 3b (SMPDL3B), which represents a potential second receptor for the drug (55). Molecular studies showed that Rituximab binding to SMPDL3B in podocytes modifies the interaction with synaptopodin and prevents cytoskeleton remodeling induced by serum of patients with FSGS (55, 56). There are also chances that other cell lineages, such as Th17, could be modified by Rituximab infusion (57). The interaction between anti-CD20 and circulating Th17 may have a direct pathogenic implication (58).

Besides the clinical interest in Rituximab, a putative role of B cells in MCD was studied in the past. This hypothesis is supported by some indirect associations. A point of interest is the association of MCD with multiple myeloma discussed above (22), for which was considered a possible mechanism linked to the charge of immunoglobulins. On the whole, the key question of the role of B cells in MCD seems today of renewed interest but needs further study and discussion.

## Mechanisms 3. experimental models of proteinuria

It should be emphasized that a reliable animal model of MCD has not been described yet. The main difference is the abrupt onset of massive proteinuria in human MCD that is almost absent in animal models. Another important characteristic of experimental models is that initial minimal lesions usually evolve to histological alterations typical of focal glomerulosclerosis. Therefore, it appears that studies in experimental models offer the opportunity to elucidate mechanisms of progression rather than of acute renal damage. Slow and constant increment of proteinuria would emerge as a progression factor in many of the descriptions below. The discordances between human MCD and models of proteinuria raise the main question of why MCD may maintain a normal glomerular structure in spite of podocyte effacement and heavy proteinuria. Brief persistence of proteinuria due to sensitivity to treatments and/or the possibility that protective factors occur in human MCD emerge as possible explanations but they remain uniquely as potential issues for future studies. LPS nephropathy is different from other models in terms of timing of proteinuria, that is typically transient, while renal lesions develop in spite of normal urinalysis. With this in mind, we consider the description of experimental models of proteinuria (more than of MCD) useful to facilitate comprehension of the overall problem of the pathogenesis of non-immunologic renal lesions.

### Puromycin and adriamycin nephrosis

Puromycin Aminonucleoside (PAN) was the first molecule utilized for inducing nephrotic syndrome in rats. It causes loss of glomerular basement membrane (GBM) polyanions and proteinuria within a few days from injection (59). PAN is partially metabolized into hypoxantine in glomeruli by adenosine deaminase, purine nucleoside phosphorylase and xanthine oxidase, which suggests the implication of a metabolic pathway ending with ${\text{O}}_{2}^{-}$ formation (60). However, free radical scavengers did not modify PAN toxicity *in vivo* and in podocytes *in vitro* (60, 61) suggesting the existence of parallel toxic pathways. Recent studies showed that, in glomeruli, PAN upregulates B7-1, a molecule known to induce foot process effacement and disruption of the slit-diaphragm barrier. Adriamycin (ADR) is an anthracycline antibiotic that has pleiotropic effects on podocytes. It may alter DNA, induces lipid peroxidation (62), and/or directly alters cell cytoskeleton (63); it also produces depletion of mitochondrial DNA (64). ADR was introduced in experimental nephrology in 1982 (65), when it was observed that its infusion in rats determined rapid loss of GBM polyanions, proteinuria, and initial minimal glomerular changes. In mice, sensitivity to ADR was linked with maintenance of a functioning mitochondrial DNA system (66). It is of interest that ADR nephropathy occurred only in specific inbred strains in association with mutations of Prkdc gene encoding a protein critical for DNA repair (66). *In vitro* experiments suggest that free radicals are the main effectors of the toxic effect induced by the drug on podocytes and *in vivo* inhibition of proteinuria by free radical scavengers supports this concept (67). For the above reasons, ADR was considered as a mixed model of nephrosis induced by oxidants in which susceptibility to mitochondrial DNA damage plays a role (66). MCD is the starting pathological feature whereas FSGS develops over days and weeks of stable proteinuria. Overall, PAN and ADR were described in view of a possible parallel mechanism of renal damage: even if the participation of oxidants in PAN is supported by the biochemistry of metabolites, concerns still exist due to lack of inhibitory effects of anti-oxidants. B7-1 may represent the target of PAN action on podocytes (68) and as it has proved to be induced by LPS in a transient model of proteinuria (see below).

### Buffalo/Mna

Buffalo/Mna rats were first studied as a genetic model of thymoma with associated myasthenia (69, 70). Only subsequently it was found that Buffalo/Mna rats at 8 weeks of age developed proteinuria, associated with nephrotic syndrome at advanced stages, and renal lesions typical of FSGS (71). Based on segregation analysis, FSGS was initially considered genetically inherited on a recessive basis and two genes at chromosome 13 were identified as determining susceptibility (72). Further experiments clearly showed the existence of extra-renal factors correlated with a Th2 cytokine profile (39, 73). On this basis, Buffalo/Mna nephrosis was utilized to reinforce the view on immunologic factors as cause of proteinuria in conditions of genetically mediated susceptibility (40).

### Lipopolysaccharide (LPS) nephropathy

LPS is an inducer of transient proteinuria, typically beginning after 24 h from injection and lasting for 72 h; after this period, proteinuria normalizes. Renal lesions are instead progressive and after a first phase characterized by minimal lesions, FSGS develops and becomes evident after 1–2 weeks (47). LPS nephropathy is of particular interest for the evaluation of the immunomodulatory mechanism of proteinuria, principally linked to B7-1 (68). This aspect will be discussed in a dedicated section.

### Overexpression and knockout of single molecules

In the following sections, several animal models overexpressing single molecules or having undergone specific genes silencing are presented and discussed. These transgenic and knockout models represent a fascinating evolution. Their limit is that single factors or cytokines that are studied in these models are probably a part of a multi-factorial system causing proteinuria and therefore single factors *per se* play a limited role. This is demonstrated by the fact that proteinuria caused by foot process effacement is the unequivocal consequence in almost all cases. Another potential limit of these models is that they do not mirror the *in vivo* situation in terms of quantitative changes that are usually massive.

## Mechanisms 4. oxidants

Oxidants represent a potential hit for proteinuria. They are generated by polymorphonucleates interacting with bacteria or viruses and could explain why MCD is often determined by or follows an infectious episode. Animal models support oxidant implication. As already pointed out, Adriamycin and Puromycin Aminonucleoside, two molecules that have historically been utilized to induce non-immunologic proteinuria in rats, are, in fact, oxidants (60, 67).

The bulk of evidence suggesting an implication of oxidants in MCD derives from studies on the generation of oxidants by polymorphonuclear cells obtained from patients with MCD (49), or from studies on the status of anti-oxidant systems during proteinuria. Generation of *ex vivo* oxidants has already been discussed in relation to Tregs and apyrase, (49). The status of the anti-oxidant system in MCD is directly correlated with loss of albumin in urine. It is known that albumin is the most important anti-oxidant buffer in blood since chloramines, that are the product of the interaction between ${\text{O}}_{2}^{-}$ and HCO_3_, are extremely reactive with the unique free sulfhydryl group of albumin (located at the 34 Cys of the sequence). The interaction of chloramines with albumin induces the formation of a sulfonic acid. Therefore, oxidized albumin contains no free sulfhydril residue and has a modified electric charge owing to the addition of an acidic residue. Musante et al. (74) demonstrated the presence of massive amounts of oxidized albumin in active nephrotic syndrome and, considering its reduced concentration in the serum of patients with MCD, these authors proposed that massive oxidation of albumin implies that oxidants are active in MCD and that the reduced buffering system implicates reduced defense. Taken together, these two mechanisms concur to proteinuria and/or have a negative role in it.

The oxidant connection seems an interesting interpretation of events leading to proteinuria in MCD. However, for their own nature, data from *ex vivo* experiments and observations on oxidized albumin should be read with caution. We need data more directly correlated with the evolution of this disease in humans, showing in particular a positive effect of anti-oxidant therapies in nephrotic syndrome. We also need more data on the role of potentially anti-oxidant substances such as adenosine that should modify the balance of oxidants deriving from ATP. It would be easy to carry out randomized clinical trials with anti-oxidants in association with canonical approaches: however, these studies require a large number of patients and do not seem a priority at the moment. Moreover, studies on adenosine metabolism are difficult since this molecule is rapidly degraded *in vivo*.

## Mechanisms 5. specific molecules

Several single molecules have been proposed to be involved in the pathogenesis of proteinuria/MCD and should be considered outside the general context of cells and cytokines. An attractive effect of studies demonstrating that specific stimuli acting on podocytes may induce MCD is the potential development of biomarkers and/or of new strategies based on targeted drugs. Animal models facilitate this task and several specific approaches have been developed in experimental MCD. However, we still lack a homogeneous view on the context and, above all, we need the demonstration that the same mechanisms and therapies that are active in experimental models are also effective in humans.

### Cathepsin-L

Cathepsin-L has already been mentioned in relation to cyclosporine. It is a potent endoprotease responsible of the breakdown of lysosomal proteins; its secreted form is involved in extracelluar matrix degradation (75). The actin-binding protein synaptopodin is a substrate for Cathepsin-L, having an effect on cytoskeleton organization (29). Renal Cathepsin-A is overexpressed in two animal models of proteinuria, i.e., PAN nephrosis and LPS nephropathy (76), and proteinuria is reduced through administration of the Cathepsin inhibitor E-64 (76). Similarly, Cathepsin-L knockout mice are protected against LPS nephropathy. Finally, in addition to MCD, increased podocyte expression of Cathepsin-L was reported in human conditions characterized by proteinuria including membranous and diabetic nephropathy (76). This finding suggests that this protein could represent a second hit following different stimuli, from autoantibodies to metabolic conditions.

### IL13

Wistar rats overexpressing IL13 develop ultrastructural glomerular changes that are reminiscent of MCD and support a role of IL13 in the pathogenesis of experimental MCD (41). Even though IL13 mRNA is upregulated and the production of IL13 protein by CD3^+^ cells is increased in children with MCD (34), there is some concern about the above IL13 role in human beings. Of note, high levels of IL13 characterize patients with asthma and with atopic dermatitis, two very frequent conditions never associated with proteinuria (77, 78). Several anti-IL13 drugs are now available for human use and are ready for being tested in clinical trials on asthma (79, 80) and atopic dermatitis (80) in clinical trials. The inclusion of MCD in a small pilot study could resolve the open problem of IL13 role in MCD pathogenesis.

### B7-1

B7-1, also called CD80, is a trans-membrane molecule present on the surface of both B cells and other antigen presenting cells. It is a costimulatory molecule that is activated by various stimuli via Toll-like Receptors (TLRs) (81) After activation, B7-1 may interact with CD28 in Teffs and/or with CTLA4 in Tregs, potentially determining their activation (CD28) or inhibition (CTLA4) (82, 83). In podocytes, B7-1 activation is followed by effacement of foot process and proteinuria (68). Various stimuli including oxidants, as in the case of PAN, and/or inflammatory stimuli such as LPS, may activate B7-1 in glomeruli (68). Activation of the TLRs/B7-1 axis by LPS is of interest since humans are particularly exposed to potential inflammatory triggers. The induction of B7-1 by LPS and the resulting nephropathy that mimics, in the early phase, MCD, are already been described in the section dedicated to animal models. Studies in patients with MCD are also available. Garin et al. (84, 85) first showed that urinary B7-1 levels are high in patients with active MCD compared to MCD in remission and B7-1 was also detected in glomeruli of the same patients. These results were confirmed in other populations with MCD (86), thus leading to the conclusion that high B7-1 in urine and in glomeruli is a biomarker of MCD and that MCD and FSGS patients can be differentiated on this basis. This is interesting, since in animal models (here including LPS nephropathy), there is not a clear-cut difference between minimal lesions and FSGS. Other studies documented B7-1 glomerular upregulation in patients with other diseases such as diabetic nephropathy (87) and/or in patients with post-transplant FSGS recurrence (88). This observation led to the use of Abatacept, a B7-1 inhibitor, as a specific therapeutic agent in post-transplant FSGS recurrence. However, results from studies on this molecule were discordant and did not confirm the original observation (89). Currently, B7-1 blockade is not considered a proper pharmacological approach to FSGS recurrence. Overall, data on urinary and glomerular B7-1 and, in particular, the conclusion that this marker is specific for MCD require further demonstrations. Targeted therapies with Abatacept also need further studies, especially in patients with post-transplant recurrence of proteinuria.

### CD40/CD40L

CD40 is another costimulatory molecule expressed by B cells, professional antigen presenting cells, monocytes/macrophages, platelets, and also non-immune cells such as endothelial and smooth muscle cells (90). Its ligand CD40L (transiently expressed on T cells and other non-immune cells under inflammatory conditions) may also exist in a soluble circulating form. The CD40/CD40L complex mediates, at several sites, pro-inflammatory events (91), including production of cytokines, chemoattractants, oxygen radicals, etc. CD40 is also constitutively expressed in podocytes, where activation by CD40L promotes redistribution of nephrin and increases permeability to albumin in isolated glomeruli (92, 93). Previous studies showed that, in murine models of membranous nephropathy and lupus nephritis, CD40L blockade by anti-CD40L antibodies protects from autoimmune glomerulonephritis (94, 95). In a similar vein, infusion of anti-CD40L antibodies reduced proteinuria and progression of ADR nephrosis (96). Anti-CD40 antibodies were detected in FSGS patients and their levels were found to correlate with post-transplant recurrence of the disease (97); injection of the CD40/anti-CD40 complex induced proteinuria in mice. This interesting observation suggests that in some way CD40 is linked to post-transplant recurrence of FSGS, even if its exact implication is presently unclear.

### C-Mip

C-Mip (C-maf inducing protein) is an 86 KDa protein with unclear function (98) that is scarcely detected in normal human kidney. Transgenic mice overexpressing c-Mip develop a disease resembling human MCD that suggests the role of c-Mip as an effector of the disease in human beings (99). Other observations support this possibility. Overexpression of c-Mip also occurs in oncologic patients treated with inhibitors of Vascular Endothelial Growth Factor-receptor tyrosine kinase (VEGF-TKIs), such as sorafenib and sunitinib, that are utilized as inhibitors of angiogenesis (100, 101). These patients frequently develop MCD/FSGS and c-Mip is markedly increased in podocytes of patients who develop the disease. RelA, a factor belonging to NFk-B family that represses c-Mip transcription, is down-regulated by VEGF-TKIs (102) and this could explain why MCD is induced by VEGF (103). The implication of other factors interacting with c-Mip such as Fyn, a Src kinase involved in nephrin phosphorylation (104, 105), is now under discussion since activation of the c-Mip-Fyn axis could modify nephrin phosphorylation and eventually determine alterations of the cytoskeletal architecture and proteinuria. Overall, c-Mip seems a strong candidate as a factor implicated in the pathogenesis of MCD and studies in human beings are now in progress to conclude on this possibility.

### uPA/suPAR

The plasminogen activator receptor (uPAR) pathway has been intensely discussed in the last few years. We know from physiology that uPAR assembles αvβ3integrin and activates a signal cascade modifying adhesion to extracellular matrix (106), that is functional in maintaining podocyte shape and sieving properties of glomeruli (107). Mice lacking uPAR (*PLAUR*^−/−^) are protected from developing LPS experimental proteinuria, which implies that an excess of circulating soluble uPAR (suPAR), derived from uPAR shedding from the cell surface, may be a determinant of proteinuria associated with LPS (107, 108). However, serum levels of suPAR are not increased in nephrotic patients, while high serum levels correlate with renal function (109). Apolipoproptein (APOL1) genotype has been recently proposed as third actor of the pathway involving suPAR and integrins (110), in which case G1 or G2 variants of APOL1 would have affinity for αvβ3integrin and suPAR and might cause proteinuria on a suPAR dependent basis. This finding follows the demonstration that *APOL1* is a haplotype of risk of developing glomerulosclerosis and HIV-nephropathy (111) and could explain the reason why not all subjects carrying this haplotype develop renal failure. Presently, further studies are required before concluding on suPAR pathogenetic role.

### Angiopoietin-like 4

ANGPTL4 is a glycoprotein, expressed by several tissues and organs (i.e., adipose tissue, liver, skeletal muscle, heart), that exists in two isoforms with high (pI > 8) and neutral (p = I7) isoelectric point, depending on the different contents of sialic acid. The idea of ANGPTL4 involvement in MCD derives from the observation of transgenic rats which have a generalized (aP2-ANGPTL4) or site-specific overexpression of ANGPTL4 in podocytes (NPHS2-ANGPTL4) (112). It is of interest that the nephrotic syndrome does not develop in all cases but only in mice with podocyte overexpression of ANGPTL4, having normal circulating levels of the protein. Rats overexpressing ANGPTL4 outside the kidney have instead high circulating levels of this factor but do not develop proteinuria. Circulating and glomerular ANGPTL4 are markedly high in PAN nephrosis and are reduced by 70% by glucocorticoids; reduced serum levels are not followed by resolution of proteinuria. Therefore, there are concerns about the meaning and implication of circulating ANGPTL4 in experimental nephrosis. Data in human beings are more homogeneous and follow a logical sequence: ANGPTL4 is minimally expressed in normal glomeruli, but it is upregulated in MCD (113) and serum and urine ANGPTL4 levels are high in these patients (113). Therefore, high circulating ANGPTL4 levels and high renal expression coincide in human MCD and correspond to proteinuria. A possible explanation reconciling data deriving from animal models is that ANGPTL4 may determine proteinuria acting specifically on podocytes and that a site specific effect does not necessarily mean high circulating levels. ANGPTL4 remains a strong candidate for explaining MCD both in human beings and in experimental models.

### Hemopexin

Plasma hemopexin is a glycoprotein involved in the binding and transport of free heme and iron homeostasis. It is considered an acute phase protein with anti-oxidant function (114, 115). When infused into rats, hemopexin induces reversible proteinuria supported by actin and nephrin reorganization in glomeruli (116) Studies in human MCD showed decreased titer of plasma and urine hemopexin and increased activity exclusively during disease relapse. These changes were ascribed to an altered configuration of hemopexin in relapsing MCD (117). Conclusions on hemopexin are still far from being reached and its implication in MCD seems, at the moment, a secondary effect.

## Overview and conclusive remarks

The definition of molecular and cellular mechanisms for proteinuria in MCD has been the topic of many studies in the last few decades (Table 3) and several interesting and plausible conclusions have been reached. Most data on these mechanisms are based on animal models and human studies have also been carried out. A major problem is the definition of MCD, that is based on pathology characteristics, while it is still not clear whether MCD is an autonomous disease or represents a phase of a progressive condition leading to FSGS. In animal models, the continuum from MCD to FSGS has been clearly documented, but data in human beings are of difficult interpretation and we lack a definite conclusion on this aspect. Studies showed an implication of different circulating cells and documented the role of cytokines and of other soluble factors. Based on this complex picture, the unique possible conclusion is that the pathogenesis of MCD is multifactorial and here we propose the concept that MCD is a “clinical-pathological-genetic entity” in which many molecules are involved. Indeed, it is also clear that we lack a single causative factor for human MCD. Good news come from therapeutic developments that are changing the clinical impact of the disease, shifting from a day by day approach to the administration of drugs active in the mid/long-term that are better tolerated by patients (53, 54, 118, 119). These evolutions reinforce our hope for a conclusive solution deriving from pharmacological developments.

**Table 3:** Principal fields of research on mechanisms for Minimal Change Disease.

	**References**
**Animal Models**	
-Adriamycin	(23, 24)
-Puromicin Aminonucleoside	(20–22)
-LPS	(25)
-Buffalo/Mna	(28–32)
-IL13 overexpression	(36, 41)
**Circulating Cells**	
-T4/T8	(34)
-T regs	(32, 44, 46–48, 52)
**Oxidants**	
-apyrase	(48)
-ATP	(48)
-O2 -	(21)
**Specific Molecules**	
-IL13	(36, 41)
-B7-1/CD80	(25, 67–69, 71, 72)
-CD40/CD40L	(75–79)
-C-mip	(80, 81, 86)
-uPA/suPAR	(89–92)
-Angiopoietin-like 4	(94, 95)
-Hemopexin	(98, 99)

## Ethics statement

Clinical studies cited in this article and conducted by our research group were approved by local Ethics Committee. A written informed consent was in all cases obtained from parents of patients. All clinical investigations were conducted according to the principles expressed in the Declaration of Helsinki Experiments in animals were done by our research group according to the principles expressed in the Declaration of Helsinki and were approved by the Institutional Review Board of IRCCS S. Martino (Genoa) and by the local authorities according to the legal requirements.

## Author contributions

AB and AC performed clinical observations, and GC genetic testing and correlations of some patients with MCN cited in the study. RB and GG prepared the manuscript, and endorsed the final draft submitted.

### Conflict of interest statement

The authors declare that the research was conducted in the absence of any commercial or financial relationships that could be construed as a potential conflict of interest.
